# Voluntary assisted dying: estimating life expectancy to determine eligibility

**DOI:** 10.5694/mja2.51790

**Published:** 2022-11-24

**Authors:** Sharon H Nahm, Martin R Stockler, Belinda E Kiely

**Affiliations:** ^1^ NHMRC Clinical Trials Centre, University of Sydney and Sydney Cancer Centre Sydney NSW

**Keywords:** Legislation, medical; Suicide, assisted

## Open access

Open access publishing facilitated by The University of Sydney, as part of the Wiley – The University of Sydney agreement via the Council of Australian University Librarians.

## Competing interests

No relevant disclosures.

in reply: We thank Ryan and colleagues^1^ for reiterating the challenges interpreting the life expectancy criteria in Australian laws regarding voluntary assisted dying (VAD).

Ryan and colleagues recommend a plain English interpretation, using the wording from the Victorian legislation as an example: “expected to cause death within weeks or months, not exceeding 6 months”. We agree and posit that these words mean death within 6 months is expected, and that survival beyond 6 months is not expected. Ryan and colleagues also state that “clinical judgement is the beginning and end of this criterion”. We agree with this too, but this clinical judgement about death occurring within a given period is inherently probabilistic.

Our aim was therefore to help doctors identify those patients unlikely to live beyond 6 months using our experience researching prognostication in advanced cancer.^2^ We are not advocating that the criterion regarding life expectancy should be narrowed from 6 months to 2 months. We are advocating that Australia's VAD laws should be harmonised and improved by including clearer definitions and explanations of phrases such as “expected to cause death within 6 months” to clarify how certain a doctor should be that a person requesting VAD would otherwise die within the specified time. This formulation would correspond better with how prognoses should be estimated and communicated.
